# A role for flies (Diptera) in the transmission of *Campylobacter* to broilers?

**DOI:** 10.1017/S0950268816001539

**Published:** 2016-08-15

**Authors:** A. ROYDEN, A. WEDLEY, J. Y. MERGA, S. RUSHTON, B. HALD, T. HUMPHREY, N. J. WILLIAMS

**Affiliations:** 1 Department of Epidemiology and Population Health, Institute of Infection and Global Health, University of Liverpool, Liverpool, UK; 2 School of Biology, University of Newcastle, Newcastle-upon-Tyne, UK; 3 National Food Institute, Technical University of Denmark, Søborg, Denmark

**Keywords:** Broiler chicken, *Campylobacter*, Diptera, flies, foodborne zoonoses

## Abstract

*Campylobacter* is the leading cause of bacterial diarrhoeal disease worldwide, with raw and undercooked poultry meat and products the primary source of infection. Colonization of broiler chicken flocks with *Campylobacter* has proved difficult to prevent, even with high levels of biosecurity. Dipteran flies are proven carriers of *Campylobacter* and their ingress into broiler houses may contribute to its transmission to broiler chickens. However, this has not been investigated in the UK. *Campylobacter* was cultured from 2195 flies collected from four UK broiler farms. Of flies cultured individually, 0·22% [2/902, 95% confidence interval (CI) 0–0·53] were positive by culture for *Campylobacter* spp. Additionally, 1293 flies were grouped by family and cultured in 127 batches: 4/127 (3·15%, 95% CI 0·11-6·19) from three broiler farms were positive for *Campylobacter*. Multilocus sequence typing of isolates demonstrated that the flies were carrying broiler-associated sequence types, responsible for human enteric illness. Malaise traps were used to survey the dipteran species diversity on study farms and also revealed up to 612 flies present around broiler-house ventilation inlets over a 2-h period. Therefore, despite the low prevalence of *Campylobacter* cultured from flies, the risk of transmission by this route may be high, particularly during summer when fly populations are greatest.

## INTRODUCTION

*Campylobacter* is the leading cause of human bacterial diarrhoeal disease worldwide. In the European Union (EU) alone, 214 779 cases were reported in 2013 [1]. However, due to under-reporting, this figure underestimates the true impact of the disease. For example, in the UK only one in ten cases are reported to national surveillance schemes [2]. Aside from possible complications of acute infection, campylobacteriosis can lead to a number of chronic sequelae [3]. These significant public health implications are coupled to a serious socioeconomic burden, with campylobacteriosis costing the EU 0·35 million disability-adjusted life-years and €2·4 billion per annum [4].

The majority (>90%) of cases are caused by *C. jejuni,* with the second most frequently isolated species, *C. coli*, accounting for a further 5–10% of cases [5]. Both species colonize the intestinal mucosa of food-producing animals, including poultry [3]. Raw chicken meat can have very high (>10^7^ cells per carcass) contamination levels [6] and may account for up to 70% of cases of campylobacteriosis [7]. An EU-wide survey conducted on *Campylobacter* in broiler chickens at slaughter, found 75·3% of broiler batches colonized with *Campylobacter* in the UK, Europe's largest producer of broiler meat [8]. The prevention of broiler flock colonization has become a European food safety priority.

However, *Campylobacter* colonization of broiler flocks is difficult to prevent even if good biosecurity is maintained, particularly during the summer months. Campylobacteriosis in temperate regions, such as the UK, has distinct seasonality, with the peak incidence of human cases occurring in summer [9]. This correlates with the peak in broiler *Campylobacter* colonization, which occurs in late spring and summer [10]. This peak in flock colonization remains unexplained and hypotheses include seasonal changes in climatic factors, which concurrently result in peak fly (Diptera) populations during summer months [10–12].

It has been established previously that flies are carriers of *Campylobacter* spp. and are proven vectors of other enterobacterial pathogens [13–15]. Flies act as mechanical vectors, transmitting microorganisms as fomites, and as biological vectors, multiplying pathogens within the gut [13, 16]. *Campylobacter* spp. are common in the farm environment and livestock faeces represent a major environmental source [17, 18]. Studies conducted within the poultry farm environment have demonstrated the carriage of *Campylobacter* spp. by flies [13, 19, 20]. In addition, flies have been shown to transmit *C. jejuni* to chickens under laboratory conditions [21], and molecular typing in a Danish field study strongly implicated flies in the transmission of a strain of *C. jejuni* from sheep to broilers [22]. Further, Danish studies strongly suggest that the ingress of flies into broiler houses contributes to the transmission and colonization of broiler chickens with *Campylobacter* spp. [23, 24].

While data are available from several countries to support the hypothesis that flies are implicated in the epidemiology of *Campylobacter* colonization of broiler flocks, field studies have not previously taken place in the UK and there are no data on the carriage of *Campylobacter* spp. by flies on and around UK broiler farms. Therefore, this study aimed to investigate the role of flies in the transmission of *Campylobacter* spp. to broiler chickens in the UK.

## MATERIALS AND METHODS

### Data collection 1

#### Collection and identification of flies

Flies were caught on four broiler farms within a 2·5 mile radius in North Wales, UK, from July to August 2011. The four broiler farms consisted of three or four broiler houses stocking about 26 000– 30 000 broilers per house per flock cycle. All four farms operated an all-in/all-out system, with thinning at around day 35 and a 36- to 42-day crop cycle. The houses operated a tunnel ventilation system. All four broiler farms were part of food assurance schemes requiring adherence to a number of strict biosecurity protocols, including, but not limited to, restricted and monitored access, an anteroom at the entrance to the broiler house containing a physical barrier delineating a biosecure area, widespread use of disinfectant footbaths, farm- and broiler-house-specific clothing and footwear and rigorous policies preventing the introduction and spread of disease. The four farms were in close proximity to livestock, including ruminants and horses. Live flies were collected for culture during at least one whole crop cycle on each farm, by repeat visits once or twice weekly. At each sampling event, flies were individually collected in sterile re-sealable plastic bags outside broiler houses (<10 m periphery) for 2 h. An open bag was placed into a plastic jar and the jar placed over a fly resting on a surface to capture it. The bag containing the fly was removed from the jar and transported to the laboratory for processing.

Captured flies were anaesthetized through placement at −18 °C for 10 min and identified taxonomically to family level under a microscope. Dipteran families were categorized as (i) filth flies, (ii) livestock, dung or carrion associated flies and (iii) other flies. Filth flies commonly consume and breed in excreta and carrion and include those of the families Calliphoridae, Fanniidae and Muscidae [14]. Where possible, the species of Calliphoridae, Fanniidae or Muscidae were determined, as these have previously been most highly implicated in the carriage of *Campylobacter* spp. [12, 14, 22]. Category (ii) contains families comprising a large proportion of species dependent upon animal excrement or organic matter for part of their lifecycle, i.e. mating, oviposition or feeding. The families contained in category (iii) are generally not associated with livestock, dung or carrion.

#### Bacterial isolation and identification

Following identification, each fly was individually and manually macerated for 10 s, enriched in 10 ml modified Exeter selective enrichment broth [1100 ml nutrient broth, 11 ml lysed defibrinated horse blood, *Campylobacter* enrichment supplement SV59 (Mast Group Ltd, UK) and *Campylobacter* growth supplement SV61 (Mast Group Ltd)] and incubated under microaerobic conditions at 42 °C. After 24 h and 48 h incubation, 5 *µ*l of enrichment broth was streaked onto *Campylobacter*-selective blood-free agar [modified charcoal-cefoperazone-deoxycholate agar (mCCDA)] supplemented with cefoperazone (32 mg/l) and amphotericin B (10 mg/l). These plates were incubated microaerobically at 42 °C for 48 h and up to four colonies per plate were selected based on morphology and streaked onto Columbia agar containing 5% (v/v) defibrinated horse blood. Plates were incubated for 48 h under both microaerobic conditions at 42 °C and aerobic conditions at 37 °C, to distinguish morphologically similar *Campylobacter* and *Arcobacter* species. Four *Campylobacter* isolates were stored at −80 °C in Microbank™ vials (Pro-Lab, UK). All media were obtained from Lab M Ltd (UK) and all blood from Southern Group Labs (UK).

Genus- and species-specific polymerase chain reaction (PCR) assays were performed on the four selected suspected *Campylobacter* colonies. To extract DNA, ~10 *µ*l of bacterial cells were suspended in 300 *µ*l chelex solution [20% w/v in 100 ml Trizma HCl (Sigma-Aldrich, UK)] and heated at 95 °C for 10 min, centrifuged at 16200 ***g*** for 3 min and 50 *µ*l of the clear supernatant added to 450 *µ*l sterile distilled water. A confirmatory multiplex 16S rDNA PCR assay for *Campylobacter* and *Arcobacter* spp. [25, 26] was followed by a multiplex *C. jejuni* and *C. coli* specific PCR assay [27]. Each PCR reaction consisted of 21 *µ*l of 1·1 × (2·5 mm MgCl_2_) ReddyMix PCR Master Mix (Thermo Scientific, UK), 1 *µ*l of bovine serum albumin (Sigma-Aldrich), 0·25 *µ*l of each primer and 2 *µ*l template DNA. DNA amplification was performed for 30 cycles using an annealing temperature of 50 °C. PCR amplicons were visualized after electrophoresis on a 2% agarose gel (Alpha Laboratories, UK).

### Data collection 2

#### Collection and identification of flies

A second sampling phase was conducted on the same four broiler farms sampled in data collection 1 from June to August 2012. As described, flies were collected on farm and transported to the laboratory. Captured flies were anaesthetized with CO_2_ gas and identified taxonomically to family level. While anaesthetized, flies were grouped into batches of ~10 flies of similar dipteran families using sterile forceps.

#### Bacterial isolation and identification

Following grouping of flies into batches, microbiological culture and PCR of *Campylobacter* spp. was carried out as in data collection 1.

#### Broiler chicken flock sampling and testing for Campylobacter

One broiler chicken flock on each of the four farms was tested daily for *Campylobacter* from June to August 2012. To sample the broiler flock, one pair of disposable fabric boot socks (overshoes) was worn over rubber boots as the sampler walked 100 m inside the broiler house. Boot socks were pre-moistened with sterile physiological saline to allow maximum uptake of *Campylobacter* from the litter. Each pair of boot socks was then placed into an individual sterile re-sealable plastic bag for transport. In the laboratory, 200 ml sterile physiological saline was added to each pair of boot socks and agitated by hand to release attached matter. Boot socks and saline were left to settle for 10 min, then 1 ml clear upper fluid was transferred to a sterile 1·7 ml Eppendorf tube and centrifuged at 16200 ***g*** for 7 min. The supernatant was discarded and DNA extracted from the pellet using the Promega Genomic DNA Wizard Extraction kit (Promega, UK). Presence of *Campylobacter* was confirmed by performing PCR as in data collection 1. To confirm flock positivity following a positive PCR result, 30 swabs of excreted chicken faeces were collected from the broiler flock. Faecal swabs were streaked directly onto mCCDA and incubated microaerobically at 42 °C for 48 h. If <28 swabs were *Campylobacter*-positive by culture, a further 30 faecal swabs were collected every other day until at least 28 positive swabs were obtained. Additionally, the flock continued to be boot-sock sampled daily until *Campylobacter* had been isolated from at least 28 faecal swabs. Finally, ten *Campylobacter* isolates were randomly selected from these final 28–30 cultures to represent ten individual faecal samples. Isolates were stored at −80 °C in Microbank™ vials (Pro-Lab).

#### Multilocus sequence typing (MLST) analysis

To assess genetic diversity, MLST profiles were obtained for *C. jejuni* and *C. coli* isolates. From the four isolates stored from each *Campylobacter*-positive batch of flies and ten isolates from each *Campylobacter*-positive flock, two isolates were randomly selected for MLST from each positive batch of flies or broiler flock. These were subjected to the *Campylobacter-*specific MLST scheme as described by Miller *et al*. [28], with alternative PCR and sequencing primers used for the *tkt* locus where necessary [29]. Sequence data were analysed using ChromasPro (Technelysium Pty Ltd, Australia) and allele numbers and sequence types (STs) assigned using the *Campylobacter* MLST website (developed by Keith Jolley and sited at the University of Oxford; http://pubmlst.org/campylobacter/) [30].

#### Malaise trap survey of flies (Diptera)

Malaise traps were used for community analysis to survey the species and numbers of Diptera gaining access to the broiler houses through the ventilation inlets and to analyse the diversity of dipteran species present on the study farms. A Malaise trap is a tent-like structure, where flies and other insects fly into the tent wall and are funnelled up to the highest point and into a collection vessel. Two Malaise traps were placed perpendicular to broiler-house walls, at a right angle to the insect flight line, at each sampling event for 2 h. Seventy per cent ethanol was used in the collection vessel as a killing agent and preservative prior to identification.

## RESULTS

### Data collection 1

#### Culture of *Campylobacter* spp.

Flies were sampled on each broiler farm 6–9 times, totalling 29 sampling events on all four farms. A mean of 31 flies were caught during each sampling event. A total of 902 flies, representing 28 families, were collected and cultured individually (Supplementary Table S1). Two flies were found to be carrying *Campylobacter* spp.; a *Calliphora vomitoria* or ‘blue-bottle’ was positive for *C. lari* and a fly of the Heleomyzidae, a dipteran family associated with livestock, dung or carrion, was found to be carrying *C. coli*. The prevalence of *Campylobacter* spp. from flies was calculated to be 0·22% [2/902, 95% confidence interval (CI) 0-0·53].

### Data collection 2

#### Culture and MLST of *Campylobacter* spp.

Flies were sampled on each broiler farm 5–6 times, totalling 21 sampling events on all four farms. A mean of 62 flies were caught during each sampling event. A total of 1293 flies, representing 29 families, were collected and cultured in 127 batches with a mean of ten flies per batch (range 1–17) (Supplementary Table S2). Batch size varied due to grouping of similar families of Diptera to aid taxonomical identification of dipteran species carrying *Campylobacter* spp.

*C. jejuni* was isolated from four batches of flies from the broiler farms (4/127, 3·15%, 95% CI 0·11-6·19) (Table 1). One positive fly batch (farm D) contained only species of the family Calliphoridae, while the other three positive batches contained a variety of species from dipteran families associated with livestock, dung or carrion, including the filth-fly families, Calliphoridae, Fanniidae and Muscidae.

**Table 1.** Batches of flies (Diptera) from four broiler farms in the UK testing positive for *Campylobacter* spp. between June and August 2012 (data collection 2) and STs obtained through MLST of *Campylobacter* isolates from flies and broiler flocks

Farm	Sampling date	Flock age when flies *Campylobacter*-positive (days)	*Campylobacter* sp. isolated from flies [ST (CC)]	Flies present in positive batch	Flock age when broilers *Campylobacter*-positive (days)	*Campylobacter* sp. isolated from flock [ST (CC)] [flock age at isolation (days)]
A	August 2012	Flies *Campylobacter-*negative	Flies *Campylobacter-*negative	Flies *Campylobacter-*negative	21	*C. coli* [ST828 (CC828)] (21)
B	June 2012	27	*C. jejuni*	7 filth flies (1 Fanniidae, 1 *Muscina stabulans*, 3 *Phaonia* sp., 2 *Polietes lardarius*); 3 livestock, dung and carrion associated flies (1 Anthomyiidae, 1 Psychodidae, 1 Scatophagidae); 2 other flies (1 Dolichopodidae, 1 unidentified sp.)	21	*C. jejuni*
	(flock cycle 1)		[ST1701 (CC45)]			(ST unknown) (29)
	August 2012	Flies *Campylobacter-*negative	Flies *Campylobacter-*negative	Flies *Campylobacter-*negative	15	*C. jejuni* [ST257 (CC257)] (21)
	(flock cycle 2)					
C	June 2012	Flies *Campylobacter-*negative	Flies *Campylobacter-*negative	Flies *Campylobacter-*negative	35	*C. jejuni*
	(flock cycle 1)					(ST unknown) (37)
	July 2012 (flock cycle 2)	2	*C. jejuni* [ST137 (CC45)]	3 filth flies (1 Fanniidae, 1 Muscidae, 1 *Phaonia* sp.); 7 livestock, dung and carrion associated flies (3 Anthomyiidae, 3 Scatophagidae, 1 Sciaridae)	21	Unidentified *Campylobacter* sp.
						(ST unknown) (21)
D	July 2012	26	*C. jejuni*	10 filth flies (8 *Calliphora vicina*, 1 *Calliphora vomitoria*, 1 *Phormia terraenovae*)	13	*C. jejuni*
			[ST25 (CC45)]			[ST257 (CC257)] (15)
		26	*C. jejuni*	9 filth flies (1 *Calliphora vicina*, 5 *Fannia canicularis*, 1 *Phaonia* sp., 2 *Stomoxys calcitrans*); 1 livestock, dung and carrion associated flies (1 Scatophagidae)	13	*C. jejuni*
			[ST1701 (CC45)]			[ST257 (CC257)] (15)

ST, Sequence type; CC, clonal complex; MLST, multilocus sequence typing.

Three *C. jejuni* sequence types (STs) were identified from the four *C. jejuni*-positive batches of flies caught on three broiler farms, with ST1701 [clonal complex (CC) 45] identified on two farms (B and D). During the study period, a total of six flock cycles occurred across the four broiler farms, all of which were *Campylobacter-*positive before thinning. From these six flocks, MLST profiles were obtained for two isolates from each of three *Campylobacter-*positive flocks. These six allelic profiles revealed two STs, with both isolates from each flock identified as the same ST. The broiler flock at farm A tested positive for *C. coli* [ST828 (CC828)] at 21 days of age. However, no *Campylobacter-*positive flies were caught on this farm. On farm D, the broilers were found positive for *C. jejuni* on day 13 [ST257 (CC257)] and two batches of flies caught on day 26 were positive for two different *C. jejuni* STs of the same CC (ST25 and ST1701 of CC45). On farms B and C, two flock cycles were covered during the study. On farm B, the first flock during the study period was positive for *C. jejuni* (unknown ST) at 21 days; 6 days before *C. jejuni* [ST1701 (CC45)] was cultured from a batch of flies. The second flock was *C. jejuni*-positive [ST257 (CC257)] at 15 days; however, no *Campylobacter-*positive flies were caught during this flock cycle. During the first flock cycle of the study period on farm C, the flock was positive for *C. jejuni* (unknown ST), but no positive flies were caught. During the second flock cycle, *C. jejuni* [ST137 (CC45)] was identified in a batch of flies at 2 days and the flock was positive for an unidentified *Campylobacter* spp. at 21 days.

#### Malaise trap survey of flies (Diptera)

Two Malaise traps were set up during 20 sampling events, collecting a total of 1771 insects (class: Insecta), including 1644 flies, from 28 dipteran families (Table 2, Supplementary Table S3). The range of Diptera caught in one sampling event was 0–612.

**Table 2.** Number of insects (class: Insecta) and flies (Diptera) caught in Malaise traps on four broiler farms in the UK between June and August 2012 (data collection 2)

	Broiler farm A	Broiler farm B	Broiler farm C	Broiler farm D	Broiler farm total
Filth flies	1 (0·8)	8 (0·7)	6 (1·7)	10 (4·9)	25 (1·4)
Livestock, dung or carrion associated flies	79 (65·3)	989 (90·7)	212 (59·9)	123 (59·7)	1403 (79·2)
Other flies	34 (28·1)	61 (5·6)	75 (21·2)	46 (22·3)	216 (12·2)
Non-Diptera	7 (5·8)	32 (2·9)	61 (17·2)	27 (13·1)	127 (7·2)
Diptera (total)	114 (94·2)	1058 (97·1)	293 (82·8)	179 (86·9)	1644 (92·8)
Overall total	121 (100)	1090 (100)	354 (100)	206 (100)	1771 (100)

Values given are *n* (%).

## DISCUSSION

This is the first study to investigate the prevalence of *Campylobacter* spp. carried by flies on UK commercial broiler farms and demonstrates that flies are carriers of *Campylobacter* in this environment. The prevalence of *Campylobacter* spp. was calculated as 0·22% (2/902, 95% CI 0–0·53) from flies cultured individually in data collection 1 and 3·15% (4/127, 95% CI 0·11–6·19) from batches of flies cultured in data collection 2. Following data collection 1, it was decided to group flies into batches for logistical purposes. However, this prevented determining prevalence at the individual fly level. In data collection 1, a fly of the Heleomyzidae was found to be carrying *C. coli* and a *Calliphora vomitoria* was positive for *C. lari*. This indicates that positive batches may be the result of a single fly carrying *Campylobacter*, suggesting that the prevalence of *Campylobacter* from flies is lower than the estimated 3·15% batch prevalence. There was one minor difference in the microbiological methodology for the two data collections; in data collection 1, flies were anaesthetized by being placed at −18 °C for 10 min; however, in data collection 2, flies were anaesthetized with CO_2_ gas. This difference in anaesthetic technique is not anticipated to have affected the rate of recovery of *Campylobacter* spp. from flies. Ultimately, carriage of *Campylobacter* by flies is at low frequencies in the broiler-farm environment.

Few studies have caught flies in numbers comparable to this study. However, prevalence estimates compare favourably to European studies with similar sample sizes. Hald *et al*. [12] caught 2186 flies around five Danish broiler farms and found 31 (1·1%) to be positive for *Campylobacter* spp. However, this estimate may have been influenced by a single broiler farm, which also had a pig farm on the same site, as very few positive flies were found (<1·0%) on the four farms without additional livestock. Additionally, a Swedish study discovered 1·0% (3/291) of flies carrying *Campylobacter* spp. in the proximity of 31 broiler farms [31].

In data collection 2, isolates from *Campylobacter-*positive broiler flocks and batches of flies were typed using MLST. No direct correlation of STs was found between isolates from flies and broilers. However, the STs of three flock isolates could not be determined as amplicons were not produced with any MLST loci primers. One *C. jejuni* ST [ST257 (CC257)] and one *C. coli* ST [ST828 (CC828)] were identified in three flocks from the broiler farms. The STs identified in the broiler flocks belong to chicken-associated CCs [32]. Three *C. jejuni* STs (ST25, ST137 and ST1701) belonging to CC45 were identified in four batches of flies from three broiler farms. CC45 is associated with human enteric illness and food animal populations, particularly chicken flocks [32]. While flies were only found positive for *Campylobacter* before the broiler flock on one occasion (Table 1), this indicates that flies may transmit *Campylobacter* to humans either directly or indirectly via broiler chickens.

Transmission of *Campylobacter* spp. from flies to broilers may be successful if a source exists within a short distance of the broilers and there is a consistently high turnover rate of new flies acquiring the pathogen [33, 34]. This is viable in the UK due to the general proximity of pastured livestock to broiler farms. The four broiler farms in this study were all in close proximity to livestock, including ruminants and horses. Given the association of CC45 with food animal populations, the origin of the *C. jejuni* isolated from flies in this study may have been the surrounding livestock. Previous studies have provided evidence that flies are carriers of *Campylobacter* over short distances; Hald *et al*. [22] discovered the same macro-restriction PFGE fingerprint in 27/28 *C. jejuni* isolates from broilers, flies and sheep on one farm and Stern *et al.* [35] found *fla*A type 15 in both flies and chicken intestinal samples from one broiler farm. A Norwegian study found a clustering of broiler flocks up to 4 km apart positive for *Campylobacter* spp. in summer, which indicates the presence of factors acting on a narrow scale, such as flies [11].

The Malaise traps captured a high diversity of flies, totalling 28 families of Diptera. The Malaise traps do not confirm which fly families and species are entering broiler houses and in what frequencies. However, up to 612 flies were caught adjacent to the broiler-house ventilation inlets in just 2 h, presenting multiple opportunities for flies to enter the house. This supports the findings of other studies [12, 22, 31], which also conclude that despite the low prevalence of *Campylobacter* spp. from flies, risk of transmission is potentially high as flies are present in large numbers around broiler-house ventilation inlets. It has been estimated that the average influx of insects into a broiler house per broiler rotation is 30 728 insects (range 2233–180 300) [12]. Twenty-one per cent of these were flies, of which 20·3% were filth flies and the remaining majority composed of families that are attracted to livestock, dung or carrion. A similar distribution was seen in this study; where in total, 86·9% of flies caught in Malaise traps on broiler farms were associated with livestock, dung or carrion; however, only 1·5% were filth flies. Increased ventilation airflow during warmer weather also increases the likelihood of insects being carried passively into houses [10]. Additionally, if the *Campylobacter* prevalence from flies is compared to other vector-borne diseases, similarly low prevalences are observed, which indicates that the prevalence seen in this study may be sufficient for flies to be a regular source of colonization for the broiler flock. For example, 0·114% (*n* = 70 937) of flies were carrying *Shigella* spp. in Southwestern USA where flies were heavily implicated in the epidemiology of local cases [36].

In summary, *Campylobacter* spp. were isolated from flies caught on broiler farms and this study provides the first estimate of the prevalence of *Campylobacter* spp. from flies on UK broiler farms. This study demonstrates that flies may play a role in the transmission of *Campylobacter* to broilers. Despite the low prevalences of *Campylobacter* spp. detected from flies on broiler farms (0·22% and 3·15%), risk of transmission is high as flies are present in large numbers around broiler-house ventilation inlets. MLST demonstrated that flies commonly carry broiler-associated STs, responsible for human enteric illness. Flies threaten the microbiological safety of the food chain, with an ability to breach existing biosecurity measures and colonize broiler flocks. Future studies should aim to quantify the risk of colonization that flies present to UK broiler farms and implement strategies to reduce this risk. In Denmark, fly screens have been shown to significantly (*P* = 0·0002) reduce the prevalence of *Campylobacter-*positive flocks at slaughter [23] and the use of a similar case-control study in the UK would be beneficial in assessing the risk that flies present and the effectiveness of available control measures.

## Supplementary material

For supplementary material accompanying this paper visit https://doi.org/10.1017/S0950268816001539. click here to view supplementary material
