# Supplementary material for: A role for flies (Diptera) in the transmission of Campylobacter to broilers?
Source: Epidemiol Infect. 2016 Aug 15;144(15):3326–34. doi: 10.1017/S0950268816001539 (PMC5080666; doi:10.1017/S0950268816001539)
Supplement: Supplementary file 1 [file S0950268816001539sup001.docx]

Supplementary Table S1: Number of flies (*Diptera*) per family collected for culture of *Campylobacter* spp. outside broiler houses (<10m periphery) on four broiler farms in the UK from July to August 2011 (Data Collection 1).

|  | **Farm A** | **Farm B** | **Farm C** | **Farm D** | **Total** |  |
| --- | --- | --- | --- | --- | --- | --- |
| **Filth Flies** | | | | | | |
| ***Calliphoridae*** |  |  |  |  |  |  |
| *Calliphora vomitoria* (L.) | 4 | 9 | 6 | 29 | 48 |  |
| *Lucilia* spp. (R-D.) | 2 | 2 | 39 | 3 | 46 |  |
| *Phormia terraenovae* (R-D.) | 63 | 39 | 29 | 46 | 177 |  |
| *Pollenia rudis* (F.) | 6 | 18 | 3 | 1 | 28 |  |
| *Other* | 1 | 1 | 0 | 1 | 3 |  |
| ***Fanniidae*** |  |  |  |  |  |  |
| *Fannia canicularis* (L.) | 80 | 14 | 58 | 39 | 191 |  |
| ***Muscidae*** |  |  |  |  |  |  |
| *Hydrotaea irritans* (Fall.) | 0 | 2 | 0 | 0 | 2 |  |
| *Musca domestica* (L.) | 5 | 0 | 8 | 5 | 18 |  |
| *Muscina stabulans* (Fall.) | 1 | 6 | 17 | 51 | 75 |  |
| *Neomyia* spp. | 0 | 1 | 1 | 0 | 2 |  |
| *Stomoxys calcitrans* (L.) | 15 | 20 | 7 | 2 | 44 |  |
| *Other* | 11 | 8 | 6 | 7 | 32 |  |
| **SUBTOTAL** | **188** | **120** | **174** | **184** | **666** |  |
| **Livestock, Dung and Carrion Associated Flies** | | | | | | |
| ***Anthomyiidae*** | 4 | 9 | 6 | 31 | 50 |  |
| ***Bibionidae*** | 1 | 7 | 15 | 3 | 26 |  |
| ***Culicidae*** | 0 | 0 | 1 | 0 | 1 |  |
| ***Heleomyzidae*** | 2 | 0 | 0 | 2 | 4 |  |
| ***Mycetophilidae*** | 0 | 0 | 0 | 1 | 1 |  |
| ***Phoridae*** | 0 | 0 | 0 | 1 | 1 |  |
| ***Psychodidae*** | 1 | 0 | 1 | 4 | 6 |  |
| ***Sarcophagidae*** | 1 | 1 | 0 | 0 | 2 |  |
| ***Scatopsidae*** | 1 | 0 | 3 | 2 | 6 |  |
| ***Sciaridae*** | 0 | 0 | 0 | 3 | 3 |  |
| ***Sepsidae*** | 3 | 1 | 3 | 3 | 10 |  |
| ***Sphaeroceridae*** | 0 | 0 | 4 | 8 | 12 |  |
| ***Stratiomyidae*** | 1 | 2 | 2 | 3 | 8 |  |
| ***Syrphidae*** | 1 | 0 | 1 | 1 | 3 |  |
| ***Tabanidae*** | 0 | 0 | 1 | 0 | 1 |  |
| **SUBTOTAL** | **15** | **20** | **37** | **62** | **134** |  |
| **Other Flies** | | | | | | |
| ***Agromyzidae*** | 0 | 0 | 0 | 4 | 4 |  |
| ***Anisopodidae*** | 4 | 1 | 1 | 4 | 10 |  |
| ***Athericidae*** | 0 | 0 | 0 | 5 | 5 |  |
| ***Cecidomyiidae*** | 0 | 0 | 0 | 3 | 3 |  |
| ***Chironomidae*** | 0 | 0 | 0 | 2 | 2 |  |
| ***Dolichopodidae*** | 2 | 1 | 4 | 38 | 45 |  |
| ***Empididae*** | 0 | 0 | 1 | 0 | 1 |  |
| ***Hybotidae*** | 0 | 8 | 0 | 3 | 11 |  |
| **Other Acalyptratae** | 0 | 1 | 2 | 1 | 4 |  |
| ***Pallopteridae*** | 0 | 1 | 0 | 1 | 2 |  |
| **SUBTOTAL** | **6** | **12** | **8** | **61** | **87** |  |
|  |  |  |  |  |  |  |
| **Unidentified *Diptera*** | **4** | **3** | **2** | **6** | **15** |  |
| **TOTAL (*Diptera*)** | **213** | **155** | **221** | **313** | **902** |  |

Supplementary Table S2: Number of flies (*Diptera*) per family collected for culture of *Campylobacter* spp. outside broiler houses (<10m periphery) on four broiler farms in the UK from June to August 2012 (Data Collection 2).

|  | **Farm A** | **Farm B** | **Farm C** | **Farm D** | **Total** |  |
| --- | --- | --- | --- | --- | --- | --- |
| **Filth Flies** | | | | | | |
| ***Calliphoridae*** |  |  |  |  |  |  |
| *Calliphora vicina* (R-D.) | 1 | 0 | 2 | 15 | 18 |  |
| *Calliphora vomitoria* (L.) | 0 | 4 | 0 | 4 | 8 |  |
| *Lucilia* spp. (R-D.) | 0 | 0 | 1 | 0 | 1 |  |
| *Phormia terraenovae* (R-D.) | 3 | 86 | 10 | 1 | 100 |  |
| *Pollenia rudis* (F.) | 6 | 2 | 1 | 1 | 10 |  |
| ***Fanniidae*** |  |  |  |  |  |  |
| *Fannia canicularis* (L.) | 32 | 115 | 54 | 37 | 238 |  |
| *Other* | 3 | 3 | 10 | 3 | 19 |  |
| ***Muscidae*** |  |  |  |  |  |  |
| *Hydrotaea irritans* (Fall.) | 1 | 1 | 0 | 9 | 11 |  |
| *Musca domestica* (L.) | 0 | 5 | 0 | 1 | 6 |  |
| *Muscina stabulans* (Fall.) | 1 | 3 | 12 | 2 | 18 |  |
| *Phaonia* spp. (R-D.) | 3 | 3 | 4 | 9 | 19 |  |
| *Polietes lardarius* (F.) | 0 | 3 | 1 | 0 | 4 |  |
| *Stomoxys calcitrans* (L.) | 10 | 32 | 11 | 4 | 57 |  |
| *Other* | 2 | 0 | 1 | 3 | 6 |  |
| **SUBTOTAL** | **62** | **257** | **107** | **89** | **515** |  |
| **Livestock, Dung and Carrion Associated Flies** | | | | | | |
| ***Anthomyiidae*** | 68 | 11 | 57 | 17 | 153 |  |
| ***Ceratopogonidae*** | 1 | 0 | 2 | 2 | 5 |  |
| ***Culicidae*** | 0 | 0 | 1 | 0 | 1 |  |
| ***Drosophilidae*** | 1 | 0 | 0 | 11 | 12 |  |
| ***Heleomyzidae*** | 1 | 0 | 0 | 4 | 5 |  |
| ***Phoridae*** | 0 | 0 | 6 | 0 | 6 |  |
| ***Psychodidae*** | 1 | 1 | 4 | 3 | 9 |  |
| ***Scatophagidae*** | 187 | 68 | 121 | 9 | 385 |  |
| ***Scatopsidae*** | 1 | 0 | 0 | 5 | 6 |  |
| ***Sciaridae*** | 1 | 1 | 5 | 4 | 11 |  |
| ***Sepsidae*** | 0 | 2 | 5 | 0 | 7 |  |
| ***Sphaeroceridae*** | 3 | 17 | 2 | 5 | 27 |  |
| ***Stratiomyidae*** | 0 | 0 | 4 | 7 | 11 |  |
| ***Syrphidae*** | 0 | 0 | 0 | 1 | 1 |  |
| ***Tabanidae*** | 0 | 1 | 0 | 0 | 1 |  |
| **SUBTOTAL** | **264** | **101** | **207** | **68** | **640** |  |
| **Other Flies** | | | | | | |
| ***Anisopodidae*** | 2 | 0 | 0 | 10 | 12 |  |
| ***Cecidomyiidae*** | 0 | 5 | 1 | 1 | 7 |  |
| ***Chaoboridae*** | 0 | 0 | 1 | 0 | 1 |  |
| ***Chironomidae*** | 6 | 2 | 15 | 8 | 31 |  |
| ***Chloropidae*** | 0 | 1 | 0 | 0 | 1 |  |
| ***Dolichopodidae*** | 3 | 6 | 6 | 39 | 54 |  |
| ***Hybotidae*** | 0 | 0 | 3 | 4 | 7 |  |
| ***Lonchopteridae*** | 10 | 1 | 0 | 0 | 11 |  |
| **Other Acalyptratae** | 0 | 1 | 0 | 0 | 1 |  |
| ***Pallopteridae*** | 1 | 0 | 0 | 3 | 4 |  |
| ***Rhagionidae*** | 0 | 1 | 4 | 4 | 9 |  |
| **SUBTOTAL** | **22** | **17** | **30** | **69** | **138** |  |
|  |  |  |  |  |  |  |
| **TOTAL (*Diptera*)** | **348** | **375** | **344** | **226** | **1293** |  |

Supplementary Table S3: Number of flies (*Diptera*) per family captured in Malaise traps erected outside broiler houses (<10m periphery) on four broiler farms in the UK from June to August 2012 (Data Collection 2).

|  | **Farm A** | **Farm B** | **Farm C** | **Farm D** | **Total** |  |
| --- | --- | --- | --- | --- | --- | --- |
| **Filth Flies** | | | | | | |
| ***Calliphoridae*** |  |  |  |  |  |  |
| *Lucilia* spp. (R-D.) | 0 | 0 | 1 | 1 | 2 |  |
| *Pollenia rudis* (F.) | 0 | 0 | 0 | 1 | 1 |  |
| ***Fanniidae*** | 0 | 0 | 0 | 1 | 1 |  |
| ***Muscidae*** |  |  |  |  |  |  |
| *Hydrotaea irritans* (Fall.) | 0 | 6 | 3 | 5 | 14 |  |
| *Musca domestica* (L.) | 0 | 0 | 1 | 0 | 1 |  |
| *Muscina stabulans* (Fall.) | 1 | 0 | 1 | 0 | 2 |  |
| *Phaonia* spp. (R-D.) | 0 | 0 | 0 | 2 | 2 |  |
| *Stomoxys calcitrans* (L.) | 0 | 1 | 0 | 0 | 1 |  |
| *Other* | 0 | 1 | 0 | 0 | 1 |  |
| **SUBTOTAL** | **1** | **8** | **6** | **10** | **25** |  |
| **Livestock, Dung and Carrion Associated Flies** | | | | | | |
| ***Anthomyiidae*** | 6 | 5 | 52 | 9 | 72 |  |
| ***Ceratopogonidae*** | 1 | 4 | 6 | 0 | 11 |  |
| ***Drosophilidae*** | 0 | 1 | 10 | 5 | 16 |  |
| ***Heleomyzidae*** | 0 | 0 | 0 | 1 | 1 |  |
| ***Phoridae*** | 1 | 10 | 11 | 1 | 23 |  |
| ***Psychodidae*** | 2 | 25 | 6 | 4 | 37 |  |
| ***Sepsidae*** | 1 | 25 | 3 | 2 | 31 |  |
| ***Scatophagidae*** | 40 | 70 | 41 | 20 | 171 |  |
| ***Scatopsidae*** | 0 | 3 | 2 | 10 | 15 |  |
| ***Sciaridae*** | 12 | 40 | 33 | 33 | 118 |  |
| ***Sphaeroceridae*** | 16 | 800 | 45 | 36 | 897 |  |
| ***Stratiomyidae*** | 0 | 5 | 3 | 2 | 10 |  |
| ***Syrphidae*** | 0 | 1 | 0 | 0 | 1 |  |
| **SUBTOTAL** | **79** | **989** | **212** | **123** | **1403** |  |
| **Other Flies** | | | | | | |
| ***Agromyzidae*** | 0 | 2 | 0 | 3 | 5 |  |
| ***Cecidomyiidae*** | 6 | 23 | 16 | 4 | 49 |  |
| ***Chironomidae*** | 24 | 26 | 43 | 31 | 124 |  |
| ***Chloropidae*** | 0 | 2 | 1 | 1 | 4 |  |
| ***Dolichopodidae*** | 1 | 2 | 8 | 3 | 14 |  |
| ***Empididae*** | 0 | 2 | 4 | 2 | 8 |  |
| ***Hybotidae*** | 0 | 0 | 0 | 1 | 1 |  |
| ***Lonchopteridae*** | 3 | 0 | 0 | 0 | 3 |  |
| **Other Acalyptratae** | 0 | 1 | 2 | 0 | 3 |  |
| ***Pallopteridae*** | 0 | 3 | 0 | 0 | 3 |  |
| ***Periscelididae*** | 0 | 0 | 1 | 0 | 1 |  |
| ***Sciomyzidae*** | 0 | 0 | 0 | 1 | 1 |  |
| **SUBTOTAL** | **34** | **61** | **75** | **46** | **216** |  |
|  |  |  |  |  |  |  |
| **Non-*Diptera* (Other Insecta)** | 7 | 32 | 61 | 27 | 127 |  |
| **TOTAL (*Diptera*)** | 114 | 1058 | 293 | 179 | 1644 |  |
| **TOTAL (Overall)** | **121** | **1090** | **354** | **206** | **1771** |  |
